# Post-hoc simulation study of computerized adaptive testing for the Korean Medical Licensing Examination

**DOI:** 10.3352/jeehp.2018.15.14

**Published:** 2018-05-17

**Authors:** Dong Gi Seo, Jeongwook Choi

**Affiliations:** Department of Psychology, College of Social Science, Hallym University, Chuncheon, Korea; Hallym University, Korea

**Keywords:** Algorithms, Computers, Korea, Logistic models, Research design

## Abstract

**Purpose:**

Computerized adaptive testing (CAT) has been adopted in licensing examinations because it improves the efficiency and accuracy of the tests, as shown in many studies. This simulation study investigated CAT scoring and item selection methods for the Korean Medical Licensing Examination (KMLE).

**Methods:**

This study used a post-hoc (real data) simulation design. The item bank used in this study included all items from the January 2017 KMLE. All CAT algorithms for this study were implemented using the ‘catR’ package in the R program.

**Results:**

In terms of accuracy, the Rasch and 2-parametric logistic (PL) models performed better than the 3PL model. The ‘modal a posteriori’ and ‘expected a posterior’ methods provided more accurate estimates than maximum likelihood estimation or weighted likelihood estimation. Furthermore, maximum posterior weighted information and minimum expected posterior variance performed better than other item selection methods. In terms of efficiency, the Rasch model is recommended to reduce test length.

**Conclusion:**

Before implementing live CAT, a simulation study should be performed under varied test conditions. Based on a simulation study, and based on the results, specific scoring and item selection methods should be predetermined.

## Introduction

The Korean Medical Licensing Examination (KMLE) was established to ensure the adequate preparation of medical professionals. Cognitive ability in the context of the KMLE can be defined as possessing the knowledge, skills, abilities, and judgment necessary to provide effective medical care. The KMLE extends beyond knowledge recall, and assess examinees’ capacity to perform the higher mental processes of reasoning, remembering, understanding, problemsolving, and decision-making. Further, the KMLE represents an important measurement of suitability for the dynamic hospital setting, which requires quick processing and decision making. As a new KMLE is being developed, the Korean Health Personnel Licensing Examination Institute is preparing test centers and launching a committee on computerized adaptive testing (CAT) [1]. However, CAT has not been previously been implemented for any high-stakes examinations, such as licensing examinations. Therefore, the objective of this study was to suggest the most appropriate scoring method and item selection method for implementing CAT on the future KMLE.

As a CAT scoring method, Wang and Vispoel [2] recommended the Bayesian estimator over the maximum likelihood estimation (MLE). However, the Bayesian estimator was found to be more severely biased. Weiss and McBride [3] were concerned that the Bayesian methods may become more biased as *θ* approaches the extremes due to regression toward the mean of the prior [4]. Therefore, this study evaluated whether this previous research can be generalized to the setting of CAT for the KMLE. In CAT item selection research, a number of new methods have been proposed to account for uncertainty in $\hat{\theta }$ during item selection. These include maximum information and Kullback-Leibler (K-L) information-based item selection procedures. Veerkamp and Berger [5] as well as van der Linden [6] proposed interval-based item selection procedures. Simulation studies found that these methods provided improvements in terms of bias and root mean square error (RMSE) in the early stages of CAT. However, the benefits disappeared as the test length increased to 10 items and *θ* became more accurately estimated. The following item response theory (IRT) models were applied in this study: the Rasch model using only the item difficulty parameters, the 2-parameter IRT model using item difficulty and discrimination parameters, and the 3-parameter IRT model using item difficulty, discrimination, and guessing parameters. The 3-parameter logistic (3PL) model is defined as:

(1) Pu=1θ=c+1-cexpDaθ-b1+expDaθ-b

where *a* is the discrimination parameter, *b* is the difficulty parameter, and the *c* is the pseudo-guessing parameter. The *c* parameter equals the probability of an examinee of maximally low ability (*θ*) obtaining a correct response due to guessing. Thus, *c* is also the lower asymptote of the item response function. The inclusion of the *c* parameter affects the location of *a* and *b* on the *θ* scale.

This study explored the accuracy and efficiency of CAT across 4 scoring methods, 6 item selection methods, and 3 IRT models.

## Methods

### Ethical statement

This study was exempted from the requirement to obtain informed consent by the Institutional Review Board of Hallym University (HIRB-2015-047), because there was no identifiable content in the data.

### Study design

This study was an analysis of simulated data estimated by a real test program.

### Simulation test design

There are 3 types of research designs in the literature on CAT. First, a Monte Carlo simulation study simulates both person and item parameters to generate responses under specific conditions. Second, a post-hoc simulation study uses item parameters from a real item bank for CAT. The *θ* estimated by CAT is compared with the true *θ* estimated by the full set of items to evaluate the recovery of the true *θ* under different conditions. Third, a live CAT study is performed with real candidates in a practical test setting. This study was designed as a post-hoc simulation test using item parameters from real KMLE data. A conventional test was previously taken to measure candidates’ scores, and the complete data-matrix was then used in this CAT simulation study. Because the true *θ* is not known, a post-hoc simulation is a typical design to evaluate the effect of varying different CAT algorithms under specific conditions. All CAT algorithms for this study were implemented using the ‘catR’ package [7] in the R program [8].

### Real data

A simulation study was conducted using data collected from the KMLE administered in January 2017. The KMLE contained 8 different content area that included the different numbers of items (Table 1). Each content area possessed unique items that candidates were required to learn as content objectives. Table 1 shows the content specification, including the number of items. Based on the content specification, the content-balanced procedure proposed by Kingsbury and Zara [9] was applied to this simulation study. The CAT algorithm randomly selected the content area for the first 5 items and then the content area that was most divergent from the targeted percentage was selected next to meet the test plan. Once the content area was determined by a greatest-divergence procedure, the algorithm randomly selected the items in that content area with the probability of a correct response that was closest to the target probability of 60%. The desired content coverage of the KMLE was specified as the percentage of the test items that came from each of the content areas in the test plan (Table 1). The number of examinees in the real data was 3,259 and the number of items was 360. The real data are available in Supplement 1.

The KMLE was administered at 5 different test centers in Korea by the Korea Health Personnel Licensing Examination Institute as a paper-based test. The candidates took around 4 hours to complete the exam. The item types were multiple-choice with 1 best answer and R-type. The response data consisted of 0 or 1. Table 2 presents descriptive statistics for the item parameters. The KMLE was designed to be appropriate for screening low-ability examinees.

### Technical information

Four scoring methods were used to calibrate examinees’ scores in this CAT simulation study. The first scoring method was maximum likelihood (ML). The goal of ML is to find an estimate of *θ* that maximizes the likelihood of observing the response pattern given the items administered. ML does not work if examinee does not have all 0s or all 1s in his/her response pattern. When the response pattern is nonmixed, the likelihood function will still be a monotonically increasing function, like the item response curve. This problem has been addressed by combining ML with other estimation methods. The second method was the weighted likelihood estimation (WLE). Based on the ML estimator, as *n* becomes large, the bias approaches zero. In applied testing circumstances, *n* is not arbitrarily large. Thus, bias will not asymptotically zero. In order to correct the bias of the ML estimator, Warm proposed the WLE method to adjust the first derivative of the log likelihood [10]. The third method was the modal a posteriori (MAP) estimation. This method involves estimating the value of *θ* that maximizes the likelihood of observing the response pattern given the prior distribution. Iterative procedures such as the Newton-Raphson are commonly used to locate the maximum of the posterior. The fourth method was expected a posterior (EAP) estimation. The EAP method involves finding the expected value of the posterior by using quadrature weight corresponding to the prior distribution. If the normal distribution is used, then the weights equal the area under the normal distribution contained between the quadrature points [11].

This CAT study evaluated 6 item selection methods. The first was the maximum Fisher information (MFI) method, which selects the item that provides the MFI at $\hat{\theta }$ [12]. Fisher information provides the amount of measurement precision at a given $\hat{\theta }$. The item that provides maximum information at the current $\hat{\theta }$ best measures the current ability during CAT administration. The second method is maximum likelihood weighted information [5], which weights Fisher information by the likelihood function to take into account uncertainty about $\hat{\theta }$. The third method was maximum posterior weighted information (MPWI) [6], which finds the maximum information by weighting the information function by the posterior distribution. Therefore, the MPWI method selects the next item that provides the most posterior-weighted information in CAT. The fourth method was maximum expected information (MEI), which examines the observed information at each of the predicted $\hat{\theta }$ in terms of whether a correct or incorrect response was assigned. The MEI method selects the next item that provides the MEI in CAT. The fifth method was minimum expected posterior variance (MEPV), which selects the item that minimizes the posterior variance when each item is administered [13]. After the average of the posterior variance of the given responses is calculated for the remaining items, the MEPV method selects the next item with the smallest average posterior variance. The sixth method was K-L information, which provides global information as a candidate take an item [14]. K-L method selects the next item that provides greater discrimination between current *θ* and $\hat{\theta }$ as an item is administered.

The CAT was terminated at a cut score (-1.96) with a variablelength set of items selected from a pool of 360 KMLE items. CAT was continued until the candidate’s cognitive ability was deemed significantly above or below the passing cut score (95% confidence interval), which was based on the 2014 standard setting of the KMLE [15], or the candidate completed the maximum number of items (50).

The DETECT value was used to examine the extent of the multidimensional simple structure of the KMLE [16]. An exploratory and confirmatory DETECT analysis can be conducted using the ‘sirt’ package in the R program [8]. The confirmatory DETECT value was less than 0.1 when the 8 content areas were assumed to be 8 dimensions in the KMLE. As a result, content-balancing in CAT could consider the KMLE to have 8 dimensions.

### Statistics

In order to assess how well the true θ is recovered by CAT, several statistics have been proposed in the CAT literature. A statistic commonly used in the CAT literature is bias, which is defined as:

(2) $$Bias\;=\;\frac{\sum_{i=1}^{N}\left({\hat{\theta }}_{i\left(All\right)}-{\hat{\theta }}_{i\left(CAT\right)}\right)}{N}$$

where *N* is the number of examinees in the study (*i*= each individual).

Bias is averaged across examinees in a simulation study by computing the mean of bias values across those examinees.

The RMSE is computed by taking the square of bias and then taking the square root of the result, and has the advantage of being in the same scale as *θ*. It is defined as:

(3) $$RMSE\;=\;\sqrt{\frac{\sum_{i}^{N}{\left({\hat{\theta }}_{i\left(All\right)}-{\hat{\theta }}_{i\left(CAT\right)}\right)}^{2}}{N}}$$

The correlation statistic was provided to evaluate the recovery of the true *θ* by CAT. Finally, the efficiency of CAT was evaluated by averaging the number of items administered in CAT under each condition.

### A sample of the R code is shown below

R code [Rasch model, EAP scoring, MFI item selection case]

require(ltm)

require(irtoys)

require(catR)

setwd(“H:\CAT_simulation_2018\Analysis”)

responses < - read.table(“data2017.txt”, header= F)

items <- read.table(“medical_items_2017.csv”, header=T, sep=”,”)

res < - as.matrix(responses[,-1])

p.rasch < - est(res, model= ”1PL”,rasch= TRUE, engine= ”ltm”)

B.Rasch < - p.rasch$est

rasch< -cbind(B.Rasch,items[,^2^])

theta.eap.est < - eap(res, B.Rasch, qu= normal.qu())

EMT< -rasch

theta.gen< -theta.eap.est[,^1^]

Item_Para< -data.frame(cbind(rasch[,1:3],1,rasch[,^4^]))

colnames(Item_Para)< -list(“a”,”b”,”c”,”d”,”group”)

Item_Para$control[Item_Para$group= = ”1”] < - “A”

Item_Para$control[Item_Para$group= = ”2”] < - “B”

Item_Para$control[Item_Para$group= = ”3”] < - “C”

Item_Para$control[Item_Para$group= = ”4”] < - “D”

Item_Para$control[Item_Para$group= = ”5”] < - “E”

Item_Para$control[Item_Para$group= = ”6”] < - “F”

Item_Para$control[Item_Para$group= = ”7”] < - “G”

Item_Para$control[Item_Para$group= = ”8”] < - “H”

########## CAT Constraint ###########################

Params<-Item_Para[,-5]

start1= list(seed= NA,nrItems= 5,theta=0,startSelect= ”MFI”)

test1< -list(method= ”ML”,itemSelect= ”MFI”)

final2< -list(method= ”EAP”)

stop1< -list(rule= c(“classification”,”length”), thr= c(-1.96,50), alpha= 0.001)

cbList< - list(names= c(“A”, “B”, “C”, “D”, “E”, “F”,”G”,”H”),

props= c(0.125, 0.125, 0.125, 0.069, 0.42,0.056,0.56,0.017))

res1<- simulateRespondents(theta.gen,Params,responsesMatrix=res, start= start1,test= test1, stop= stop1,

final= final2, cbControl= cbList, save.output= TRUE,

output= c(“H:/CAT_simulation_2018/Analysis/”,”out”,”csv”) )

cbind(res1$bias,res1$RMSE, res1$correlation,res1$testLength)

## Results

Table 3 summarizes the average values of bias, RMSE, the correlation coefficient, and the number of items administered under various CAT conditions. The overall correlation coefficients ranged from 0.49 to 0.57 in the Rasch model, and from 0.81 to 0.86 in the 2PL model, and from 0.29 to 0.58 in the 3PL model. The overall RMSE ranged from 0.49 to 0.86 in the Rasch model, from 0.51 to 0.89 in the 2PL model, and from 2.35 to 3.67 in the 3PL model. The overall bias ranged from 0.05 to 0.33 in the Rasch model, from -0.01 to 0.29 in the 2PL model, from -1.87 to -0.06 in the 3PL model. Therefore, the Rasch and 2PL model performed better than the 3PL model. Overall, the recovery of the true *θ* using CAT was not good under any conditions. The reason for this is that the CAT was terminated early under all conditions because all the KMLE items were very easy and the cut-score was very low (-1.96).

For these specific conditions (easy test and low cut-score), CAT using Rasch or the 2PL model provided somewhat more accurate scores across all conditions. CAT using 3PL model overestimated candidates’ scores across all conditions. In terms of scoring method, MAP and EAP methods provided more accurate and stable scores than the MLE and WLE methods, as has been found in previous studies. An interesting finding of this study was that the WLE method showed less bias in 3PL model than the other scoring methods.

MPWI and MEPV provided more accurate scores more than the other item selection methods. All item selection methods showed less bias when the WLE scoring methods was used.

For CAT efficiency, the Rasch model was preferred to other IRT models, and the number of items administered was similar across the 4 scoring methods (on average, approximately 22 items per CAT session). In contrast to the results for accuracy, the MPWI and MEPV item selection methods showed less efficiency than other item selection methods because the CAT algorithm was designed to trade off efficiency against accuracy. The raw data are available in Supplement 1.

## Discussion

This study explored several CAT scoring and item selection methods for the KMLE. A fixed-form test artificially increases score variability due to random variability. Thus, the increased variability in test scores results in a lower correlation of test scores with other predicted scores [3]. Therefore, scores determined by a fixed-form test will show low correlations with real performance scores in practical settings. However, test scores based on CAT provide essentially unbiased scores regardless of the candidates’ ability level. Since this aspect of CAT makes candidates’ scores reliable, a CAT platform has been adopted for many licensing and certification examinations. Thus, to adopt CAT for the KMLE, the scoring and item selection methods should be evaluated in a realistic CAT setting.

As described in previous research, CAT was designed to consider the efficiency and accuracy of measurements and these factors in turn depend the circumstances of an examination. Therefore, simulation studies considering different test conditions should be performed before the practical implementation of live CAT. Since the results of CAT depend on the examination conditions (different cut scores or different test difficulty), the scoring and item selection methods under specific examination conditions should be determined before implementation.

As with any other studies, this study has some limitations. First, the KMLE currently in use assumes that the test items measure a single dominant latent trait, even if the test items are constructed as 8 dimensions. It is not always practical to assume that a test measures only a single trait. Many test batteries in educational and psychological fields are designed to measure multidimensional traits, rather than a single latent trait. For example, since KMLE has found to have 8 underlying dimensions through a practice analysis, the KMLE should provide 8 latent trait scores. If multidimensional data are modeled as unidimensional, the unidimensional item parameter estimates may measure only 1 direction of the latent traits, and the model will not fit the data well [17]. Content balancing of CAT, however, was used to consider multidimensional data in this study. Second, since the KMLE consisted of very easy items, and the cut-score was very low, CAT terminated at a very early stage. The results of this study were based on a particular implementation of CAT with this limited item bank, and therefore cannot be generalized to every all licensing examinations. More simulation studies of the KMLE should investigate different termination criteria and different cut-scores in future research. Third, conditional RMSE and conditional bias are more meaningful in Monte Carlo simulations; however, this study used average RMSE and bias because post-hoc simulations are based on real data with real item parameters and a real ability distribution. Fourth, this study did not evaluate the pool utilization and item exposure rate. Future research should evaluate the pool utilization and item exposure rate after determining scoring and item selection methods in the early period of CAT implementation.

In conclusion, this study evaluated several scoring and item selection methods that could be used for the adoption of CAT for the future KMLE. Based on our results, the 2PL model is suggested, with MAP or EAP for the scoring method and MPWI or MEPV for the item selection method to classify candidates as showing or not showing mastery. In terms of efficiency, CAT with the Rasch model terminated at an early stage across all scoring and item selection methods. Overall, CAT with the Rasch model performed relatively well compared to other IRT models in terms of both accuracy and efficiency.

## Figures and Tables

**Table 1.** Content specification of the Korean Medical Licensing Examination administered in January 2017

Content area	No. of items (%)
A	45 (12.5)
B	45 (12.5)
C	45 (12.5)
D	25 (6.9)
E	154 (42.8)
F	20 (5.6)
G	20 (5.6)
H	6 (1.7)
Total	360 (100.0)

**Table 2.** Descriptive statistics of item parameters from the Korean Medical Licensing Examination) administered in January 2017

Stats.	Percent correct	Item-total correlation	Estimated correct ratio	b-parameters
Mean	72.12	0.187	74.98	-1.28
Standard deviation	24.21	0.099	11.56	1.71
Median	79.4	0.18	80	-1.30

**Table 3.** Accuracy and efficiency of CAT for the KMLE (cut score=-1.96)

Criteria	IRT model	Scoring methods/item selection methods																							
		MLE						MAP						EAP						WLE					
		MFI	MLWI	MPWI	MEI	MEPV	K-L	MFI	MLWI	MPWI	MEI	MEPV	K-L	MFI	MLWI	MPWI	MEI	MEPV	K-L	MFI	MLWI	MPWI	MEI	MEPV	K-L
Correlation	Rasch	0.53	0.50	0.51	0.49	0.51	0.51	0.56	0.54	0.55	0.56	0.55	0.55	0.57	0.52	0.56	0.54	0.56	0.53	0.51	0.52	0.50	0.51	0.53	0.53
	2PLM	0.81	0.82	0.84	0.83	0.84	0.82	0.84	0.85	0.86	0.85	0.86	0.85	0.84	0.86	0.86	0.85	0.86	0.86	0.82	0.83	0.85	0.83	0.84	0.82
	3PLM	0.31	0.34	0.46	0.32	0.49	0.34	0.35	0.34	0.53	0.36	0.53	0.39	0.33	0.36	0.58	0.34	0.55	0.39	0.29	0.33	0.47	0.30	0.44	0.36
RMSE	Rasch	0.84	0.80	0.78	0.86	0.77	0.86	0.50	0.51	0.50	0.50	0.50	0.50	0.50	0.52	0.50	0.52	0.50	0.49	0.80	0.80	0.69	0.79	0.70	0.78
	2PLM	0.89	0.84	0.77	0.86	0.78	0.86	0.55	0.53	0.52	0.53	0.53	0.53	0.54	0.52	0.51	0.53	0.52	0.52	0.83	0.77	0.69	0.79	0.71	0.80
	3PLM	3.67	3.54	3.01	3.59	3.01	3.66	3.06	2.93	2.35	2.98	2.42	3.03	2.84	2.85	2.36	2.92	2.39	2.95	3.64	3.56	2.99	3.62	3.07	3.60
Bias	Rasch	0.31	0.28	0.29	0.30	0.27	0.33	0.07	0.06	0.07	0.05	0.06	0.06	0.08	0.08	0.09	0.09	0.07	0.07	0.27	0.27	0.21	0.25	0.22	0.27
	2PLM	0.29	0.27	0.23	0.28	0.24	0.28	-0.04	-0.04	-0.04	-0.04	-0.05	-0.05	-0.01	-0.03	-0.02	-0.03	-0.01	-0.02	0.21	0.17	0.13	0.20	0.14	0.19
	3PLM	-0.40	-0.06	-0.29	-0.29	-0.53	-0.63	-1.84	-1.59	-1.40	-1.78	-1.48	-1.86	-1.61	-1.64	-1.58	-1.78	-1.54	-1.87	-0.45	-0.27	-0.42	-0.47	-0.51	-0.73
No. of items	Rasch	15.55	15.51	14.96	15.57	15.15	15.40	15.46	15.48	15.09	15.63	15.23	15.54	15.41	15.27	15.02	15.42	15.17	15.47	15.45	15.42	15.06	15.54	15.06	15.39
administered	2PLM	30.27	30.35	30.70	30.20	30.40	30.08	30.17	30.37	30.54	30.20	30.38	30.15	29.99	30.51	30.76	30.36	30.36	30.30	30.20	30.43	30.59	30.24	30.43	30.11
	3PLM	17.67	17.07	23.83	17.46	25.30	19.66	18.18	17.73	24.45	17.92	25.14	19.88	17.25	18.35	26.54	18.46	25.82	20.36	17.45	17.53	24.37	17.80	24.84	19.54

CAT, computerized adaptive testing; KMLE, Korean Medical Licensing Examination; IRT, item response theory; MLE, maximum likelihood estimation; MAP, modal a posteriori; EAP, expected a posteriori; WLE, weighted likelihood estimation; MFI, maximum Fisher information; MLWI, maximum likelihood weighted information; MPWI, maximum posterior weighted information; MEI, maximum expected information; MEPV, minimum expected posterior variance; K-L, Kullback-Leibler; 2PLM, 2-parameter logistic model; 3PLM, 3-parameter logistic model; RMSE, root mean square error.

## Supplementary materials

Supplement 1. Data files are available from https://doi.org/10.7910/DVN/PETWZF Item responses were arranged based on 8 content areas as follows: A: items numbers 1-45 (45 items); B: 46-90 (45); C: 91-135 (45); D: 136-160 (25); E: 161-314 (154); F: 315-334 (20); G: 335-354 (20); and H: 354-350 (6).

Supplement 2. Audio recording of the abstract.
