# Funding information of the article entitled “Post-hoc simulation study of computerized adaptive testing for the Korean Medical Licensing Examination”

**DOI:** 10.3352/jeehp.2018.15.27

**Published:** 2018-12-04

**Authors:** Dong Gi Seo, Jeongwook Choi

**Affiliations:** Department of Psychology, College of Social Science, Hallym University, Chuncheon, Korea; Hallym University, Korea

We add funding information to the published article entitled “Posthoc simulation study of computerized adaptive testing for the Korean Medical Licensing Examination” [1] as follows. This funding description was omitted at the time of submission. We apologize funding institute for late description of this invaluable support.
